# Aggravation of fibrin deposition and microthrombus formation within the graft during kidney transplantation

**DOI:** 10.1038/s41598-021-97629-1

**Published:** 2021-09-23

**Authors:** Tamar A. J. van den Berg, Marius C. van den Heuvel, Janneke Wiersema-Buist, Jelle Adelmeijer, Gertrude J. Nieuwenhuijs-Moeke, Ton Lisman, Stephan J. L. Bakker, Harry van Goor, TransplantLines Investigators, J. H. Annema-de Jong, S. J. L. Bakker, S. P. Berger, J. Blokzijl, F. A. J. A. Bodewes, M. T. de Boer, K. Damman, M. H. De Borst, A. Diepstra, G. Dijkstra, R. M. Douwes, M. F. Eisenga, M. E. Erasmus, C. T. Gan, A. W. Gomes Neto, H. Grootjans, E. Hak, M. R. Heiner-Fokkema, B. G. Hepkema, F. Klont, T. J. Knobbe, D. Kremer, H. G. D. Leuvenink, W. S. Lexmond, V. E. de Meijer, H. G. M. Niesters, L. J. van Pelt, R. A. Pol, R. J. Porte, A. V. Ranchor, J. S. F. Sanders, J. C. Schutten, M. J. Siebelink, R. H. J. A. Slart, J. C. Swarte, W. Timens, D. J. Touw, M. C. van den Heuvel, C. van Leer-Buter, M. van Londen, E. A. M. Verschuuren, M. J. Vos, R. K. Weersma, Robert A. Pol

**Affiliations:** 1 Department of Surgery, University Medical Center Groningen, University of Groningen, P.O. Box 30 001, 9700 RB Groningen, The Netherlands; 2 Surgical Research Laboratory, Department of Surgery, University Medical Center Groningen, University of Groningen, Groningen, The Netherlands; 3 Department of Pathology and Medical Biology, University Medical Center Groningen, University of Groningen, Groningen, The Netherlands; 4 Department of Anesthesiology, University Medical Center Groningen, University of Groningen, Groningen, The Netherlands; 5 Division of Nephrology, Department of Internal Medicine, University Medical Center Groningen, University of Groningen, Groningen, The Netherlands; 6 Department of Health Sciences, University of Groningen, Groningen, The Netherlands; 7 Department of Gastroenterology and Hepatology, University Medical Center Groningen, University of Groningen, Groningen, The Netherlands; 8 Department of Pediatrics, University Medical Center Groningen, University of Groningen, Groningen, The Netherlands; 9 Department of Cardiology, University Medical Center Groningen, University of Groningen, Groningen, The Netherlands; 10 Department of Thoracic Surgery, University Medical Center Groningen, University of Groningen, Groningen, The Netherlands; 11 Department of Pulmonary Diseases and Tuberculosis, University Medical Center Groningen, University of Groningen, Groningen, The Netherlands; 12 Department of Pharmacy, University Medical Center Groningen, University of Groningen, Groningen, The Netherlands; 13 Laboratory Medicine, University Medical Center Groningen, University of Groningen, Groningen, The Netherlands; 14 Department of Medical Microbiology and Infection Prevention, University Medical Center Groningen, University of Groningen, Groningen, The Netherlands; 15 Cohort and Biobank Coordination Hub, University Medical Center Groningen, University of Groningen, Groningen, The Netherlands; 16 Groningen Transplant Center, University Medical Center Groningen, University of Groningen, Groningen, The Netherlands; 17 Department of Nuclear Medicine & Molecular Imaging, University Medical Center Groningen, University of Groningen, Groningen, The Netherlands

**Keywords:** Nephrology, Kidney, Renal replacement therapy

## Abstract

In kidney transplantation, microthrombi and fibrin deposition may lead to local perfusion disorders and subsequently poor initial graft function. Microthrombi are often regarded as donor-derived. However, the incidence, time of development, and potential difference between living donor kidneys (LDK) and deceased donor kidneys(DDK), remains unclear. Two open-needle biopsies, taken at preimplantation and after reperfusion, were obtained from 17 LDK and 28 DDK transplanted between 2005 and 2008. Paraffin-embedded sections were immunohistochemically stained with anti-fibrinogen antibody. Fibrin deposition intensity in peritubular capillaries(PTC) and glomeruli was categorized as negative, weak, moderate or strong and the number of microthrombi/mm^2^ was quantified. Reperfusion biopsies showed more fibrin deposition (20% to 100% moderate/strong, p < 0.001) and more microthrombi/mm^2^ (0.97 ± 1.12 vs. 0.28 ± 0.53, p < 0.01) than preimplantation biopsies. In addition, more microthrombi/mm^2^ (0.38 ± 0.61 vs. 0.09 ± 0.22, p = 0.02) and stronger fibrin intensity in glomeruli (28% vs. 0%, p < 0.01) and PTC (14% vs. 0%, p = 0.02) were observed in preimplantation DDK than LDK biopsies. After reperfusion, microthrombi/mm^2^ were comparable (p = 0.23) for LDK (0.09 ± 0.22 to 0.76 ± 0.49, p = 0.03) and DDK (0.38 ± 0.61 to 0.90 ± 1.11, p = 0.07). Upon reperfusion, there is an aggravation of microthrombus formation and fibrin deposition within the graft. The prominent increase of microthrombi in LDK indicates that they are not merely donor-derived.

## Introduction

It is well known in kidney transplantation, that microthrombi can be observed in preimplantation biopsies of kidney grafts. However, it is unclear whether the donor-derived microthrombi persist or disappear or if new microthrombi arise when the graft is reperfused. Disappearance is rather unlikely, since ischemia/reperfusion injury (IRI), which is inevitable in transplantation and is associated with a procoagulatory response, potentially enhances formation of microthrombi and deposition of fibrin. IRI induces a series of interactions between the microvasculature, tubular epithelium, and infiltrating inflammatory cells, leading to the activation of the coagulation cascade, the complement system and accumulation of fibrin(ogen) in the peritubular capillaries (PTC) of the kidney^1^. Fibrinogen, an acute phase protein, is upregulated in case of injury or inflammation^2^. When converted to fibrin within the kidney, it provides an accumulation site for platelets and red blood cells, facilitating the formation of thrombi, which subsequently can lead to local perfusion disorders and poor initial kidney graft function^3,4^.

In deceased organ donors, it has been shown that hemostasis is activated and fibrinolysis dysregulated^3^. Activation of hemostasis with subsequent microthrombus formation in deceased organ donors may be one of the reasons living donor kidneys (LDK) are of better quality than deceased donor kidneys (DDK). Despite the identification of microthrombi in preimplantation biopsies, little is known about the actual incidence, time of development and potential differences between donor types. In addition, it appears that administration of heparin to the recipient prior to reperfusion leads to less fibrin formation, reflected by lower D-dimer levels^5^. Intervening with unfractionated heparin or other antithrombotic agents might prevent extra formation of fibrin and subsequently microthrombi.

The primary aim of the current study was to investigate whether the amount of fibrin deposition and microthrombi in kidney graft biopsies changes over the course of transplantation. We further investigated whether fibrin deposition and microthrombus formation is associated with donor type or intraoperative use of heparin.

## Materials and methods

All biopsies and patient data were obtained from the TransplantLines biobank (NCT03272841), a prospective cohort study and biobank that includes all types of solid organ transplant recipients as well as (living) organ donors. The study was approved by both the TransplantLines board and the institutional ethical review board (METc UMC Groningen 2014/077). Patient data were processed and stored according to the Declaration of Helsinki. Clinical and research activities followed the principles of the Declaration of Istanbul on Organ Trafficking and Transplant Tourism.

Two open-needle biopsies were obtained from 45 kidneys (28 DDK and 17 LDK), transplanted between 2005 and 2008 after static cold storage; one prior to vascular anastomosis (preimplantation) and one 30 min after reperfusion (reperfusion). Biopsies were embedded in paraffin and stored at -80 °C. After procurement, all kidneys were flushed and perfused with cold University of Wisconsin perfusate (ViaSpan, DuPont, Wilmington, NC, USA; Belzer UW, Bridge to life, Columbia SC, USA). According to EuroTransplant protocol DBD donors received 20,000 international units (IU) of heparin prior to systemic flush. DCD donors or living kidney donors were not given any form of antithrombotic prophylaxis. The transplant procedure was performed according to local protocol which was published in detail before^6^. Per protocol, preemptively transplanted recipients, in contrast to dialysis-dependent recipients, were given 5000 IU of unfractionated heparin prior to reperfusion. Post-reperfusion analyses were performed without these nine recipients, as the effect of heparin on factors IIa and Xa in the coagulation cascade would interfere with the results. Delayed graft function (DGF) was defined as recipients receiving hemodialysis within 7 days of transplantation. Estimated glomerular filtration rate (eGFR) at 3, 6 and 12 months was calculated using the CKD-Epi formula.

### Immunohistochemical studies

**Figure 1:**
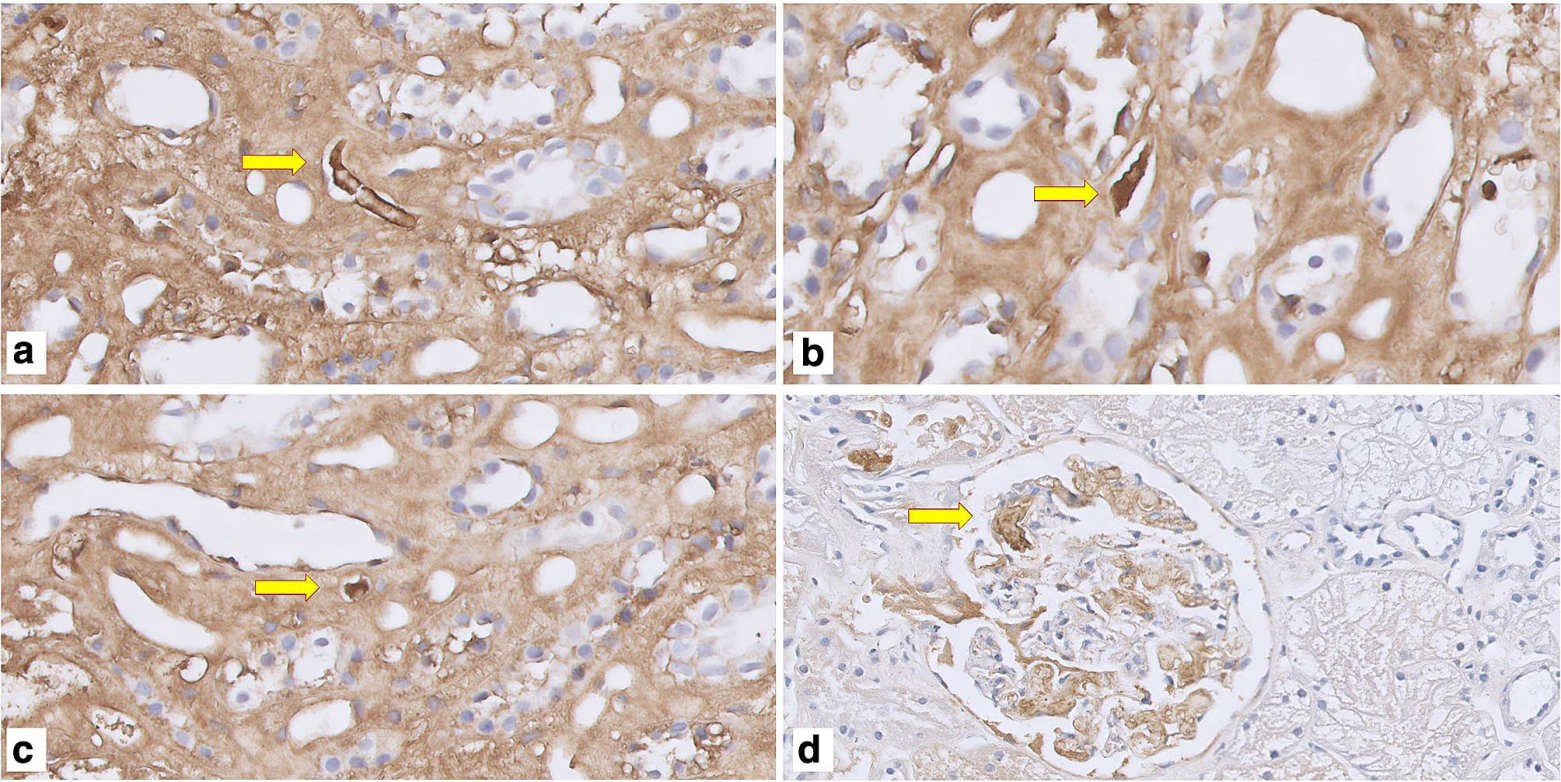
Examples of microthrombi in kidney biopsies immunohistochemically stained with and antibody against fibrin. (**a–c**) Microthrombus in peritubular capillary of reperfusion biopsy; (**d**) microthrombus in glomerular capillary in preimplantation biopsy.

**Figure 2:**
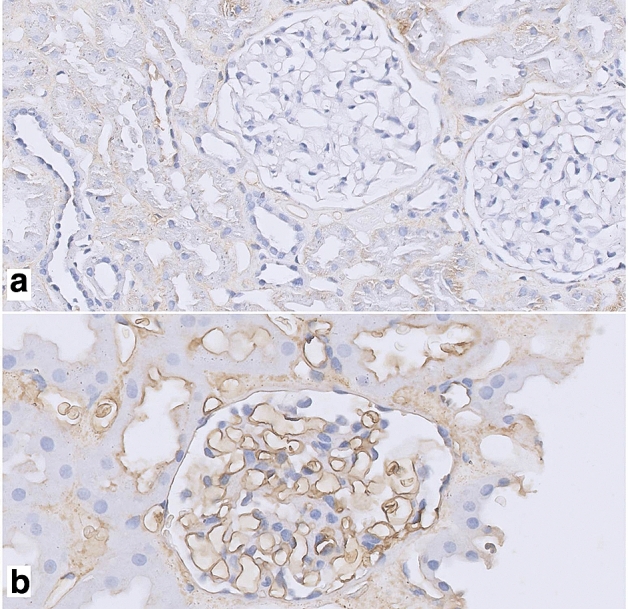
Fibrin stain intensity differences of the glomerular and PTC endothelium between preimplantation and reperfusion biopsies (IHC stain). **(a)** Example of the most observed intensity in preimplantation biopsies ( ×40  magnification); **(b)** example of most observed intensity in reperfusion biopsies (×60  magnification).

**Figure 3:**
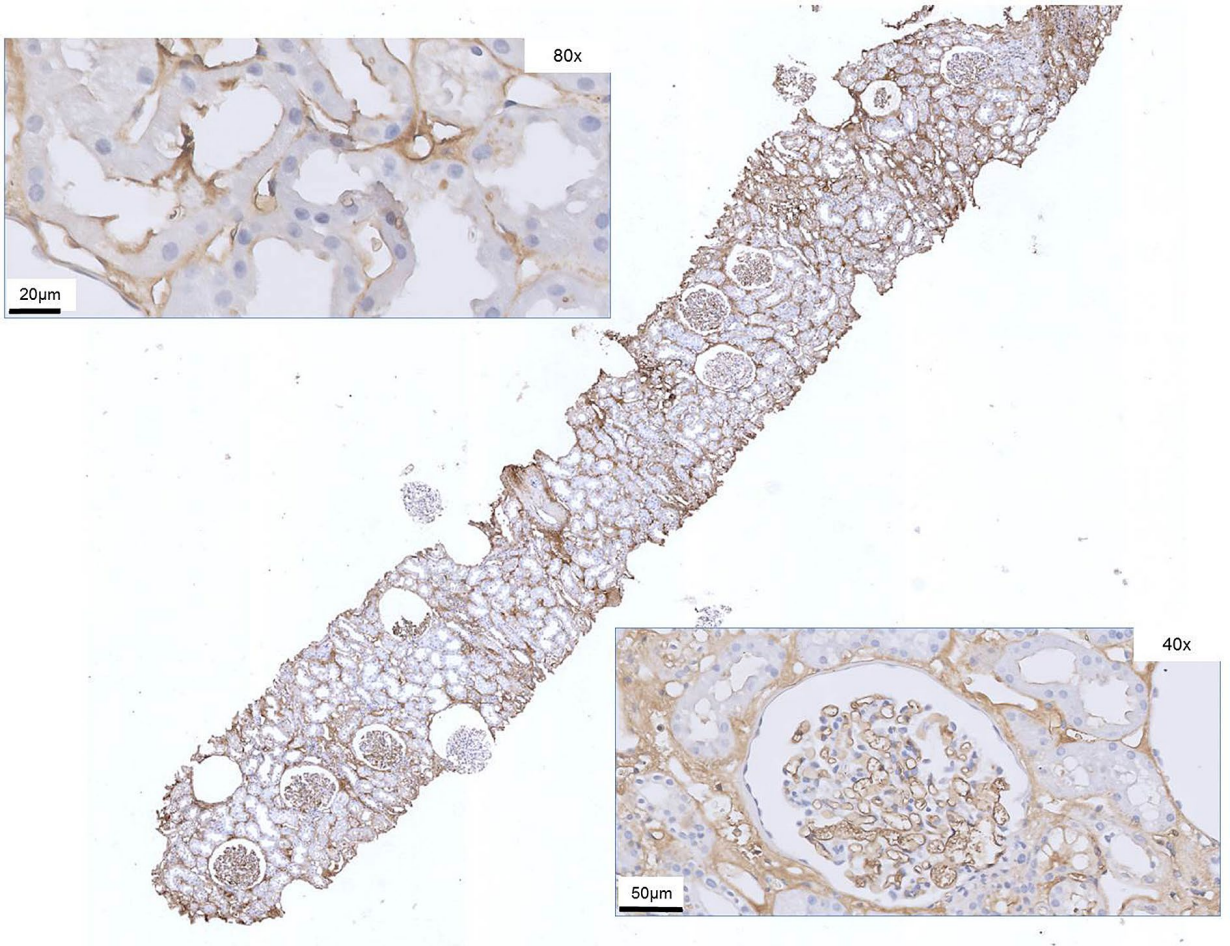
Kidney biopsy core (IHC stain with antibody against fibrin). Magnified peritubular capillaries with staining of endothelial cells (top left) and magnified glomerulus with generalized deposition of fibrin on endothelial lining (bottom right).

Microthrombi and fibrin deposition were assessed in 3μ paraffin-embedded preimplantation and post-reperfusion biopsy sections. Sections were deparaffinized with xylene and brought to 70% ethanol. Antigen retrieval was performed with proteinase K 0.1% and an endoblock using 0.3% H_2_O_2_. The sections were incubated at room temperature for 60 min with a polyclonal rabbit anti-human fibrinogen antibody (1:750, A0080; Dako, Glostrup, Denmark). Diaminobenzidine (Dako) was used as the chromogen substrate and hematoxylin as counterstain. Slides were then digitally stored using a digital slide scanner (Hamamatsu Nanozoomer HT2.0, Hamamatsu Photonics K.K., Japan) with 40× magnification and analyzed using Aperio Imagescope v12.1 (Leica Biosystems Imaging, Inc. USA). Microthrombus was defined as a cluster of fibrin threads with or without trapped red blood cells occluding parts or the whole of peritubular capillaries or glomerular capillaries (Fig. 1). Scoring was performed by a trained nephropathologist, blinded for donor type or timepoint of the biopsies. Microthrombi were scored when observed in glomeruli and/or PTC in a semi-quantitative matter. To correct for the size of the biopsy, we quantified the number of microthrombi per surface area (microthrombi/mm^2^), which was traced by hand using Aperio Imagescope. In addition, fibrin staining intensity of endothelium of peritubular capillary walls and glomeruli was assessed (Fig. 2). Generalized fibrin deposition intensity of endothelium of glomerular capillaries and PTC was graded from 0 to 3, with 0 as negative, 1 weak, 2 moderate and 3 as strong staining, according to a previously described method (Fig. 3)^7^. Additional staining was performed with Martius Scarlet Blue (MSB) stain for demonstration of fibrin, as described previously^5^.

### Statistical analysis

Descriptive statistics are presented as mean ± standard deviation (SD) or median with interquartile range (IQR) for continuous variables, depending on variable distribution. Outcomes are presented as mean with 95% confidence interval (CI). Tests of significance were two-tailed, and statistical significance is set at p < 0.05. The paired t-test or Wilcoxon matched pairs signed rank test were used to compare matched data. The Mann Whitney U-test was used to compare groups with non-normal distribution. Differences between kidneys of recipients with and without platelet aggregation inhibitor (PAI) use were evaluated by Fisher’s exact test. Associations between DGF and microthrombi were analyzed using Mann Whitney U-test. To determine the correlation between ischemia times, kidney function and microthrombi development, a Spearman correlation analysis was conducted. The results were presented by reporting Spearman’s ρ coefficient and corresponding p-values. Statistical analyses were performed using the Statistical Package for the Social Sciences (SPSS v23; IBM Corp, Armonk, NY, USA) and GraphPad Prism v5.04 (GraphPad Software Inc., La Jolla, CA, USA). Graphs were created using Graphpad Prism.

### Institutional review board statement

All biopsies and patient data were obtained from the TransplantLines biobank (NCT03272841), a prospective cohort study and biobank that includes all types of solid organ transplant recipients as well as (living) organ donors. The study was approved by both the TransplantLines board and the institutional ethical review board (METc 2014/077). Patient data were processed and stored according to the Declaration of Helsinki. Clinical and research activities followed the principles of the Declaration of Istanbul on Organ Trafficking and Transplant Tourism.

### Informed consent statement

Informed consent was obtained from all subjects involved in the TransplantLines biobank.

## Results

### Baseline characteristics

Baseline characteristics and relevant intraoperative variables of the total cohort are shown in Table 1. Seventeen kidneys were derived from living donors and 28 from deceased donors. Median age of both donors and recipients was 50 ± 14 years. Eleven recipients received single antiplatelet therapy (carbasalate calcium, n = 10; clopidogrel, n = 1) which were continued during surgery. None of the recipients were treated with anticoagulants preoperatively. Eight recipients were transplanted preemptively. These recipients all received an LDK.

**Table 1:** Baseline characteristics and intraoperative variables.

Descriptives	N of total (%) or mean (± SD)
Donor type: LDK/DDK (n)	17/28
Deceased donor type: DBD/DCD (n)	14/14
Donor age (years)	50 ± 14
**Cause of death (n)**	
Subarachnoidal bleed Stroke Trauma / other	5 / 11 / 6 / 6
Recipient age (years)	50 ± 14
Dialysis (months)	48 ± 27
Diaysis modality: hemodialysis/peritoneal dialysis (n)	24/13
Preemptively transplanted (n, %)	8 (18%, only in LDK group)
1st/2nd/3rd kidney transplant (n)	45/1/1
**Continued preoperative antithrombotic therapy**	
DDK recipients LDK recipients	10/28* 1/17**
**Intraoperative parameters**	
1st warm ischemic time (min)	7.6 ± 9.3
Cold ischemic time (min)	705 ± 487
2nd warm ischemic time (min)	42 ± 12
Intraoperative heparin (n)	8 (18%)

*carbasalate calcium, n = 9 and clopidogrel, n = 1

**carbasalate calcium, n = 1

### Development of fibrin deposition and microthrombi over time

There was an increased glomerular fibrin stain intensity in the reperfusion biopsies compared to the preimplantation biopsies, in which only 20% of the preimplantation biopsies were categorized as moderate/high glomerular fibrin stain which increased to 100% in the reperfusion biopsies (p < 0.001). There also was a difference in PTC fibrin intensity (9% vs. 65% resp., p < 0.001). The increased stain intensity in PTC or glomeruli was accompanied by comparable stain intensity of the interstitium (Figs. 2 and 3). In 37% of preimplantation biopsies and 42% of reperfusion biopsies there was some variance of stain intensity within the biopsy. Regarding staining of glomerular endothelium and PTC endothelium, 23 preimplantation biopsies showed no differences, 9 showed a one-category difference and 2 showed a two-category difference. In the reperfusion biopsies, 7 biopsies showed no differences, 22 reperfusion biopsies showed a one-category difference and 6 a two-category difference (moderate in glomeruli and mild in PTC). Glomerular stains were almost always more pronounced in case of a difference in categories in both preimplantation and reperfusion biopsies.

In addition, an increase in the number of microthrombi was observed upon reperfusion. In total, 16 preimplantation biopsies (38%) contained microthrombi compared to 28 reperfusion biopsies (65%, p < 0.01). This is in line with the significant increase in microthrombi/mm^2^ observed between preimplantation and reperfusion biopsies (0.28 ± 0.53 microthrombi/mm^2^ vs. 0.87 ± 1.00 microthrombi/mm^2^, p = 0.02; Table 2). Microthrombi were mainly observed in PTC in both preimplantation (93% of detected microthrombi) and reperfusion biopsies (80%). Sixteen (36%) patients experienced DGF after transplantation. One patient had primary non-function. This kidney presented 0.25 and 0.26 microthrombi/mm^2^ in the preimplantation and reperfusion biopsy respectively. During one procedure, there was a tear of the vena renalis, which was reconstructed. Postoperatively, this patient developed deep venous thrombosis reaching into the vena iliaca externa on the second day after KTx, after which transplantectomy was required. In the biopsies of this kidney, there were no microthrombi present at preimplantation, and 1.81/mm^2^ after reperfusion. There was no significant association between number of microthrombi/mm^2^ in preimplantation biopsies and DGF (p = 0.08) or in reperfusion biopsies and DGF (p = 0.71). Mean creatinin level after 3 months was 144 ± 70 µmol/L. Mean estimated glomerular filtration rate at 3 months was 53 ± 20 mL/min/1.73m^2^. There were no correlations between number of microthrombi at preimplantation or after reperfusion and eGFR after 3, 6 or 12 months (Table 3).

**Table 2:** Number of microthrombi/mm^2^ per group.

	Preimplantation timepoint	Reperfusion timepoint	P-value
Total	0.28 (0.12–0.45)	0.87 (0.53–1.21)	0.02
Living donor kidneys	0.09 (-0.04 to 0.22)	0.76 (0.35–1.17)	0.03
Deceased donor kidneys	0.38 (0.14–0.62)	0.90 (0.46–1.34)	0.07
DBD	0.47 (0.02–0.93)	0.92 (0.23–1.60	0.37
DCD	0.31 (0.09–0.53)	0.96 (0.27–1.66)	0.06

**Table 3:** Spearman correlations of ischemia times and kidney function and microthrombi development.

		MT/mm^2^ at preimplantation	P-value	MT/mm^2^ at reperfusion	P-value
Spearman’s ρ	Total warm ischemia time	0.042	0.79	0.229	0.14
	Cold ischemia time	0.193	0.22	0.075	0.63
	eGFR at 3 months	−0.275	0.10	0.336*	0.04
	eGFR at 6 months	−0.235	0.16	0.183	0.27
	eGFR at 1 year	−0.213	0.19	0.280	0.08

*p < 0.05

### Donor type differences

Recipients of an LDK had a significantly shorter mean time on dialysis prior to transplantation (21 ± 11 vs. 54 ± 16 months, p < 0.01), were transplanted preemptively more often (47% vs. 0%, p < 0.001), were less frequently on a platelet aggregation inhibitor (PAI) regimen (n = 10 vs. n = 1, p = 0.03) and cold ischemia time (CIT) of the kidney graft was significantly shorter (157 ± 26 min vs. 1038 ± 287 min, p < 0.001) compared to recipients of a DDK. Preimplantation biopsies of LDK showed significantly lower fibrin intensity in both glomeruli (0% vs. 28%, p < 0.01) and PTCs (0% vs. 14%, p = 0.02) than DDK. In the reperfusion biopsies, this difference disappeared in both the glomeruli (both at 88% high intensity, p > 0.99) and the PTCs (11% vs. 19%, p = 0.89).

**Figure 4:**
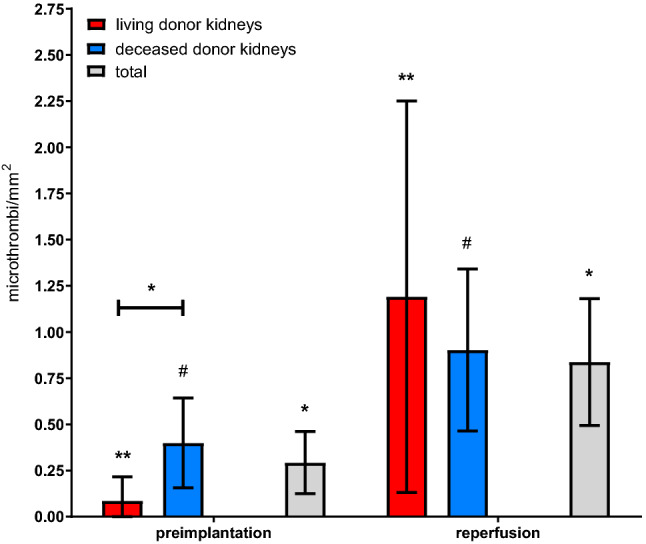
Number of microthrombi/mm^2^. Data is shown as mean with 95% confidence interval. microthrombi/mm^2^, microthrombi per square millimeter; *p = 0.02; **p = 0.03; #p = 0.07.

LDK had significantly fewer microthrombi/mm^2^ in preimplantation biopsies than DDK (0.09 ± 0.22 microthrombi/mm^2^ vs. 0.38 ± 0.61 microthrombi/mm^2^, p = 0.02, Table 2) but did show a somewhat steeper increase from preimplantation biopsies to reperfusion biopsies (0.09 ± 0.22 to 0.76 ± 0.49 microthrombi/mm^2^, p = 0.03) than DDK (0.38 ± 0.61 to 0.90 ± 1.11 microthrombi/mm^2^, p = 0.07). This resulted in a comparable mean number of microthrombi/mm^2^ between LDK and DDK in the reperfusion biopsies (p = 0.23, Fig. 4).

When stratified for deceased donor type, no differences were observed between DBD and DCD kidneys between preimplantation (0.47 ± 0.79 microthrombi/mm^2^ vs. 0.31 ± 0.37 microthrombi/mm^2^, p = 0.90) and reperfusion biopsies (0.92 ± 1.13 microthrombi/mm^2^ vs. 0.96 ± 1.15 microthrombi/mm^2^, p = 0.90, Table 2). Deceased donor characteristics are stated in Supplementary Table 1. Furthermore, no differences were observed between DDK that went to recipients who used PAI compared to recipients who did not (0.92 ± 1.16 vs. 1.06 ± 1.11 microthrombi/mm^2^ at reperfusion, p = 0.56).

### MSB stain

**Figure 5:**
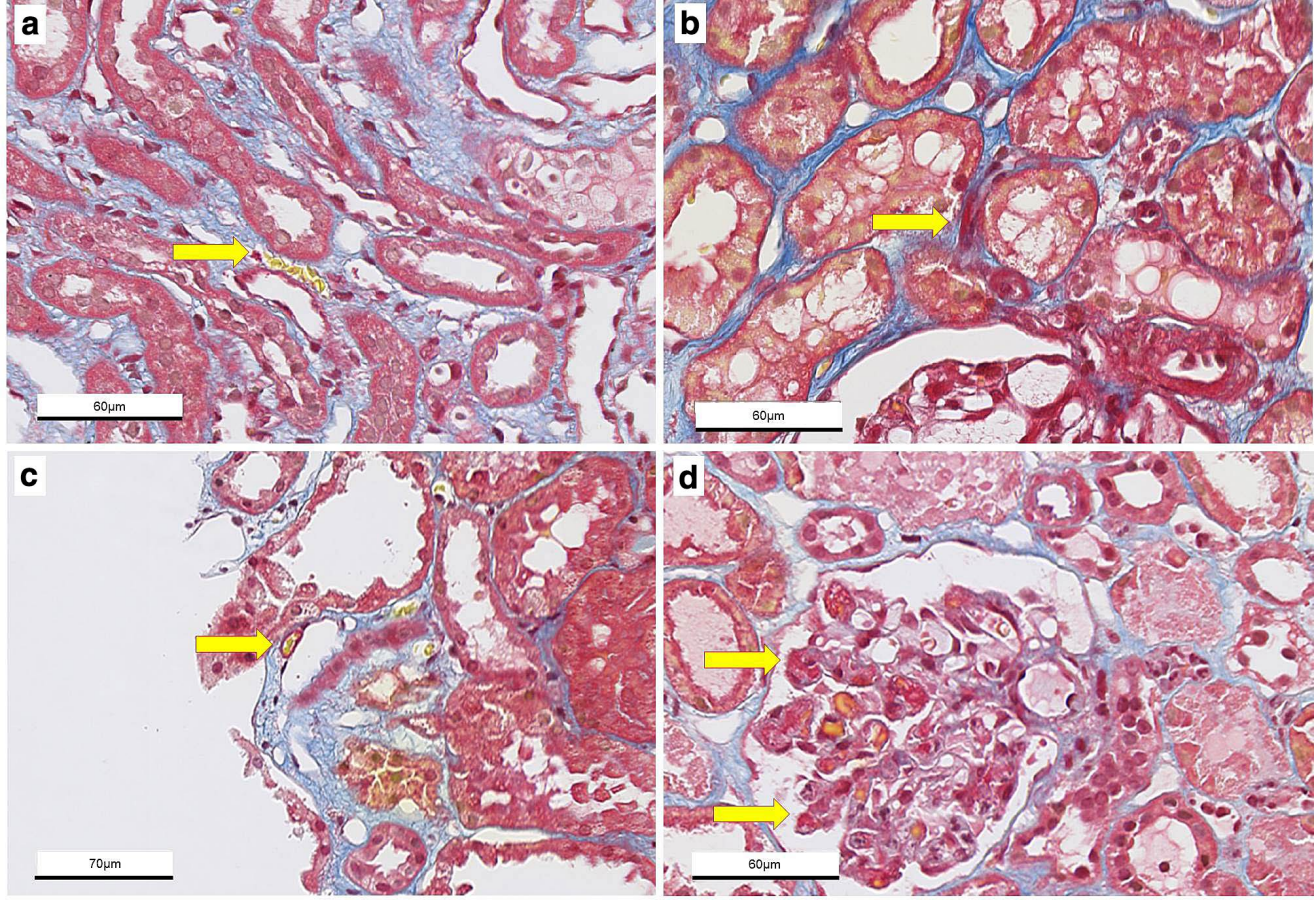
Kidney biopsies stained with Martius Scarlet Blue (MSB) stain. Fibrin stains red, collagen blue, red blood cells yellow.** (a)** Preimplantation biopsy with microthrombus in peritubular capillary, **(b)** reperfusion biopsy with microthrombus in peritubular capillary, **(c)** preimplantation biopsy with fibrin deposition on endothelial lining of peritubular capillary, **(d)** reperfusion biopsy with multiple microthrombi in glomerulus. (×40  magnification).

Microthrombi and fibrin depositions on endothelial lining of PTC were observed in the MSB stained biopsies (Fig. 5). Preimplantation biopsies showed 0.05 ± 0.24 microthrombi/mm^2^ compared to 0.15 ± 0.43 microthrombi/mm^2^ in reperfusion biopsies (p = 0.26). At preimplantation, both DDK and LDK are comparable (0.08 ± 0.29 vs. 0.00 ± 0.00 microthrombi/mm^2^, p = 0.21). DDK and LDK showed a comparable number of microthrombi/mm2 in the reperfusion biopsies (0.23 ± 0.52 vs. 0.00 ± 0.00 microthrombi/mm^2^, p = 0.06).

### Intraoperative use of heparin in the recipient and microthrombi formation

**Figure 6:**
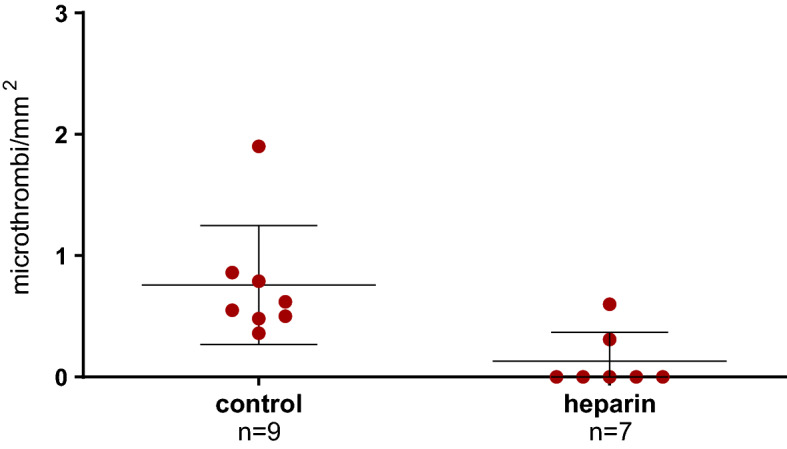
microthrombi/mm^2^ in LDK biopsies after reperfusion. Data are shown as median with 95% confidence interval. microthrombi/mm^2^, microthrombi per square millimeter; p = 0.02.

Since preemptively transplanted patients were given 5000 IU of unfractionated heparin prior to reperfusion, and as there were no recipients with a preemptive status in the deceased donor group, only LDK were stratified for heparin administration. Significantly fewer microthrombi/mm^2^ were observed in the kidneys of recipients who were given intraoperative heparin compared to controls (0.13 ± 0.24 microthrombi/mm^2^ vs. 0.76 ± 0.49 microthrombi/mm^2^, p = 0.02. Figure 6). One outlier in the LDK group was removed from the analysis due to a disagreement between the surgical report and the patient file on heparin administration. Baseline characteristics and intraoperative parameters, such as longer ischemia times or surgical complications, were comparable with the kidneys that remained in the analysis, which reduces the risk of selection bias.

## Discussion

This study shows that microthrombi not only are present in donor kidneys pre-implantation, but increase upon reperfusion and that there is a generalized increased fibrin deposition. Post-reperfusion, 0.97 microthrombi per mm^2^ biopsy tissue were observed in glomeruli and peritubular capillaries, as compared to 0.28/mm^2^ in preimplantation biopsies, and all reperfusion biopsies had a moderate to high intensity of the fibrin stain, compared to 20% in the preimplantation biopsies. In preimplantation biopsies, a higher number of microthrombi were observed in DDK compared to LDK. This difference disappeared after reperfusion, with an equal number of microthrombi/mm^2^ in both groups. Although the number of microthrombi and fibrin deposition were less pronounced in the MSB stained biopsies, the results of this stain were in line with the IHC stain. Furthermore, kidneys transplanted in recipients who were administrated 5000 IU of unfractionated heparin before clamping of the vessels showed significantly fewer microthrombi upon reperfusion than kidneys transplanted in recipients without heparin. This analysis, however, was restricted to LDK.

The higher number of microthrombi in DDK biopsies compared to LDK biopsies preimplantation, is probably due to the prolonged agonal ischemic injury seen in DCD donors, and the procoagulatory response associated with brain death in DBD donors^3,8^. The fact that the number of microthrombi/mm^2^ is equal after reperfusion in both LDK biopsies and DDK biopsies, however, indicates that the majority of microthrombi develop upon reperfusion of the graft and to a lesser extend in the donor. This finding is clinically relevant since previous studies mainly focused on formation of microthrombi in preimplantation biopsies or its association with disseminated intravascular coagulation in the donor, assuming that microthrombi are donor-derived^9–13^. Our results show that this is not the case, as LDK derived from relatively healthy donors exhibit a steady development from 0.09 microthrombi/mm^2^ in preimplantation biopsies to 1.19 microthrombi/mm^2^ in reperfusion biopsies. Extrapolation of these results to the entire cortex of the kidney would result in a considerable amount of microthrombi. These results are unexpected, as LDK are considered the perfect donor in terms of quality and graft survival, which is reflected by the low numbers of microthrombi in the preimplantation biopsies^14^. Furthermore, recipients of LDK are more often preemptively transplanted as well, contributing to a better outcome^15^. Nevertheless, we observed a greater increase in microthrombi/mm^2^ in LDK than in DDK after reperfusion. It seems unlikely that this difference is explained by a difference in thrombogenic environment, as kidneys from DDK usually experience more severe IRI. A possible explanation could be the manipulation of the kidney in hand-assisted live donor nephrectomy, which might result in bruising of the kidney. The use of a pneumoperitoneum could add to further compression. Due to the relative short time between the manipulation and the cold flush and subsequent SCS, which slows down metabolism, it could be that new microthrombi as a result of the manipulation are formed once flow is restored. However, this hypothesis does not justify systemic heparinization of the living donor^16,17^.

Reperfusion biopsies showed significantly more fibrin deposition than preimplantation biopsies. It appears that upon reperfusion an increase in accumulation of fibrin occurs, which results in a generalized and more diffuse pattern surrounding the endothelial walls of glomeruli, PTC and tubuli. This formation and accumulation of extravascular fibrinogen has been previously associated with IRI, and was found to be initiated by endothelial dysfunction after reperfusion^1,18^. These fibrin depositions form an accumulation site for microthrombi. This was also demonstrated by a recent study with kidneys undergoing normothermic machine perfusion that had formed microvascular plugs after fibrin deposition in the cortex and medulla, which caused reduced perfusion of the renal cortex. Markers of renal injury were reduced after lysis with plasminogen and tissue plasminogen activator^4,19^.

The idea of microthrombi development and activation of the coagulation cascade during renal transplantation is not new. As microthrombi are often thought to be donor-derived, several reports tried to inhibit microthrombi development by systemic heparinization of the donor. Improved kidney function or fewer surgical complications were unfortunately not achieved^16,20,21^. Although unfractionated heparin appears to be a very suitable agent to inhibit microthrombi development, by intervening at the thrombin level and inhibiting fibrinogen formation^22^, it has a short half-life^23^. Therefore, it should be administered just prior to the peak of microthrombi formation. In the current study, this peak appears to be upon reperfusion, where a pronounced increase of microthrombi in PTC was observed. This is further illustrated by the data showing that recipients who were given heparin just prior to reperfusion developed significantly fewer microthrombi in PTC. Hence, by administering heparin to the recipient at the most crucial moment of microthrombi formation, microcirculation and potentially even graft outcome could be optimized.

In this study, microthrombi were scored in both PTC and glomeruli. The occurrence of glomerular fibrin thrombi (GFT) has been investigated before, especially since GFT in preimplantation biopsies are sometimes used to accept or decline a deceased donor kidney offer. Incidences of GFT vary from 2.6% to 9.9%^11–13^. In our study GFT were present in 7.1% of the DDK. Contradictory findings regarding the influence of GFT on long-term kidney function have been reported. In two studies, GFT were shown to be a risk factor for delayed or reduced graft function in the first year after transplantation^13,24^. Two other studies nonetheless report that the majority of GFT resolve within 1 to 3 months after transplantation, and conclude that GFT do not influence kidney function after transplantation or lead to more fibrosis or glomerulosclerosis^11,25^. A recent study^12^ came to the same conclusion, although it is debatable whether their cut-off of < 50% fibrin thrombi positive glomeruli—compared to a study using 15%^26^—does not underestimate the results. In other words, it is still unclear whether a kidney with 30% of glomeruli affected by fibrin thrombi will have a function similar to that of a kidney with 10% or 45%.

It can be debated whether glomeruli are the renal structures of interest when determining the number of microthrombi in renal biopsies. The majority of microthrombi in the current study were observed in the PTC, which suggests a primary focus in these structures and less in glomerular pathogenesis. The microvasculature is especially susceptible to IRI^18^, and as PTC have limited capacity to regenerate^26^ (in contrast to tubular cells and glomeruli) they are directly associated with loss of kidney function^27^. These PTC are often neglected yet are pivotal to maintaining sufficient perfusion. Injury to the microcirculation due to endothelial cell damage may lead to permanent peritubular capillary rarefaction, which causes chronic hypoxia in these regions. This may induce transcription of fibrogenic genes like transforming growth factor-β and connective tissue growth factor together with an accumulation of α-smooth muscle actin which in the end may lead to development of IFTA (interstitial fibrosis and cortical tubular atrophy)^28^.

At reperfusion, when the renal vein and artery clamps are released, the kidney is reoxygenated after a period of ischemia. During ischemia, and in particular upon reperfusion, reactive oxygen species (ROS) are formed due to dysfunctioning of the mitochondrial respiratory chain^29^. ROS have a deleterious effect on cells due to activation of various injurious pathways through carbonylation of proteins or lipid peroxidation. At a vascular level, this will lead to swelling of endothelial cells, degradation of the cytoskeleton and loss of the glycocalix. Intercellular contact of endothelial cells is lost and subendothelial cells expressing tissue factor (TF) are exposed^28^.

Activated subendothelial TF initiates the coagulation cascade resulting in fibrin and microthrombi formation, and, in addition, IRI can also activate intravascular coagulation through activation of the complement system. Therefore, it is probable that microthrombi formation will continue after reperfusion. Although assessment of biopsies taken after the transplant procedure was not possible, due to the absence of these, previous plasma analyses of LDK have shown that coagulation markers between preemptively and non-preemptively transplanted patients are comparable, but upregulated, compared to healthy controls and remain so at least 2 h postoperatively^5^. Our current study showed that the formation of microthrombi could be reduced from 0.76 to 0.13 microthrombi/mm^2^ by administering heparin prior to reperfusion. Hypothetically, existing or developing microthrombi are lead points from where bigger thrombi can grow, as fibrin attracts platelets and activated platelets attract other platelets. By removing these lead points, possible further damage could be prevented.

This study has a few limitations that need to be addressed. First, it is a retrospective single-center study with a relatively small sample size. Unfortunately, the sample size could not be increased, since the retrieval of post-reperfusion biopsies was stopped after 2008 due to a change in protocol in our center. Additionally, to formally analyze an effect of intraoperative heparin treatment on kidney function, a larger sample size and preferably a randomized controlled trial are required which, due to the many confounding factors involved in kidney transplantation, would require a large number of patients. However, we do feel that we can draw a firm conclusion from our data due to the evident differences between the donor types, which justifies further research and even policy on heparin use. Second, there were variations in outcome between the immunohistochemical (IHC) and MSB stains. However, MSB staining has a risk of over/understaining or inadeaquate differentiation, depending on the size and maturity of the fibrin deposition^30^. The outcome of IHC staining seems more robust and has less potential for ambiguous results. Third, follow-up biopsies were not available so we were unable to determine whether a resolution or increase of microthrombi would occur in our patients. The limited sample size also prevents adequate graft survival analysis. This information would contribute to deeper insight into the pathophysiology and a future intervention.

## Conclusions

Our results show that kidney transplantation is associated with a generalized deposition of fibrin and an aggravation of microthrombi formation in peritubular capillaries and glomeruli. Although deceased donor kidneys show more preexistent microthrombi, it is mainly the living donor kidneys, which show an increase during the transplantation procedure. This increase in microthrombi and fibrin depositions indicates that microthrombi are not mainly donor-derived, but that the most significant formation occurs upon reperfusion. This might suggest a role for ischemia/reperfusion injury.

## Supplementary Information

Supplementary Information.

Supplementary Table 1.

## Data Availability

The data presented in this study are available on request from the corresponding author. The data are not publicly available for privacy reasons.
