# Supplementary material for: Aggravation of fibrin deposition and microthrombus formation within the graft during kidney transplantation
Source: Sci Rep. 2021 Sep 23;11:18937. doi: 10.1038/s41598-021-97629-1 (PMC8460629; doi:10.1038/s41598-021-97629-1)
Supplement: Supplementary file 1 — Supplementary Information. [file 41598_2021_97629_MOESM1_ESM.docx]

**Aggravation of fibrin deposition and microthrombus formation within the graft during kidney transplantation**

Tamar A.J. van den Berg 1,2*, Marius C. van den Heuvel3, Janneke Wiersema-Buist 2, Jelle Adelmeijer 2, Gertrude J. Nieuwenhuijs-Moeke 4, Ton Lisman 1,2, Stephan J.L. Bakker 5, Harry van Goor 3 and Robert A. Pol 1. TransplantLines Investigators

^1^ Department of Surgery, University of Groningen, University Medical Center Groningen, The Netherlands;

^2^ Surgical Research Laboratory, Department of Surgery, University of Groningen, University Medical Center Groningen, The Netherlands

^3^ Department of Pathology and Medical Biology, University of Groningen, University Medical Center Groningen,The Netherlands

^4^ Department of Anesthesiology, University of Groningen, University Medical Center Groningen, The Netherlands

^5^ Division of Nephrology, Department of Internal Medicine, University of Groningen, University Medical Center Groningen, The Netherlands

***Corresponding author:**

Tamar van den Berg

Department of Surgery, University Medical Center Groningen

P.O. Box 30 001

9700 RB Groningen, The Netherlands

Email: t.a.j.van.den.berg@umcg.nl

**Consortium TransplantLines**

Annema-de Jong JH^6^, Bakker SJL^5^, Berger SP^5^, Blokzijl J^7^, Bodewes FAJA^8^, de Boer MT^1^, Damman K^9^, De Borst MH^5^, Diepstra A^3^, Dijkstra G^7^, Douwes RM^5^, Eisenga MF^5^, Erasmus ME^10^, Gan CT^11^, Gomes Neto AW^5^, Grootjans H^5^, Hak E^12^, Heiner-Fokkema MR^13^, Hepkema BG^13^, Klont F^12^, Knobbe TJ^5^, Kremer D^5^, Leuvenink HGD^1,2^, Lexmond WS^8^, de Meijer VE^1^, Niesters HGM^14^, van Pelt LJ^13^, Pol RA^1^, Porte RJ^1^, Ranchor AV^6^, Sanders JSF^5^, Schutten JC^15^, Siebelink MJ^16^, Slart RHJA^17^, Swarte JC^5^, Timens W^15^, Touw DJ^12^, van den Heuvel MC^3^, van Leer-Buter C^14^, van Londen M^5^, Verschuuren EAM^11^, Vos MJ^13^, Weersma RK^7^

^1^ Department of Surgery, University of Groningen, University Medical Center Groningen, The Netherlands;
^2^ Surgical Research Laboratory, Department of Surgery, University of Groningen, University Medical Center Groningen, The Netherlands. ^3^ Department of Pathology and Medical Biology, University of Groningen, University Medical Center Groningen, The Netherlands. ^4^ Department of Anesthesiology, University of Groningen, University Medical Center Groningen, The Netherlands. ^5^ Division of Nephrology, Department of Internal Medicine, University of Groningen, University Medical Center Groningen, The Netherlands. ^6^ Department of Health Sciences, University of Groningen, The Netherlands. ^7^ Department of Gastroenterology and Hepatology, University of Groningen, University Medical Center Groningen, The Netherlands. ^8^ Department of Pediatrics, University of Groningen, University Medical Center Groningen, The Netherlands. ^9^ Department of Cardiology, University of Groningen, University Medical Center Groningen, The Netherlands. ^10^ Department of Thoracic Surgery, University of Groningen, University Medical Center Groningen, The Netherlands. ^11^ Department of Pulmonary Diseases and Tuberculosis, University Medical Center Groningen, University of Groningen, The Netherlands. ^12^ Department of Pharmacy, University of Groningen, University Medical Center Groningen, The Netherlands. ^13^ Laboratory Medicine, University of Groningen, University Medical Center Groningen, The Netherlands. ^14^ Department of Medical Microbiology and Infection Prevention, University of Groningen, University Medical Center Groningen, The Netherlands. ^15^ Cohort and Biobank Coordination Hub, University Medical Centre Groningen, University of Groningen, The Netherlands. ^16^ Groningen Transplant Center, University of Groningen, University Medical Center Groningen, The Netherlands. ^17^ Department of Nuclear Medicine & Molecular Imaging, University of Groningen, University Medical Center Groningen, The Netherlands.
