# Supplementary material for: Aggravation of fibrin deposition and microthrombus formation within the graft during kidney transplantation
Source: Sci Rep. 2021 Sep 23;11:18937. doi: 10.1038/s41598-021-97629-1 (PMC8460629; doi:10.1038/s41598-021-97629-1)
Supplement: Supplementary file 2 — Supplementary Table 1. [file 41598_2021_97629_MOESM2_ESM.docx]

**Aggravation of fibrin deposition and microthrombus formation within the graft during kidney transplantation**

Tamar A.J. van den Berg 1,2*, Marius C. van den Heuvel3, Janneke Wiersema-Buist 2, Jelle Adelmeijer 2, Gertrude J. Nieuwenhuijs-Moeke 4, Ton Lisman 1,2, Stephan J.L. Bakker 5, Harry van Goor 3 and Robert A. Pol 1. TransplantLines Investigators

^1^ Department of Surgery, University of Groningen, University Medical Center Groningen, The Netherlands;

^2^ Surgical Research Laboratory, Department of Surgery, University of Groningen, University Medical Center Groningen, The Netherlands

^3^ Department of Pathology and Medical Biology, University of Groningen, University Medical Center Groningen,The Netherlands

^4^ Department of Anesthesiology, University of Groningen, University Medical Center Groningen, The Netherlands

^5^ Division of Nephrology, Department of Internal Medicine, University of Groningen, University Medical Center Groningen, The Netherlands

***Corresponding author:**

Tamar van den Berg

Department of Surgery, University Medical Center Groningen

P.O. Box 30 001

9700 RB Groningen, The Netherlands

Email: t.a.j.van.den.berg@umcg.nl

**Keywords:** thrombosis; hemostasis; kidney transplantation; fibrin; ischemia reperfusion injury

**Supplementary Table 1:** Deceased donor characteristics

| ID | deceased donortype | Cause of death | Donor age (years) | microthrombi/mm^2^ preimplantation | microthrombi/mm^2^  after reperfusion |
| --- | --- | --- | --- | --- | --- |
| 1 | DBD | trauma capitis | 16 | 0 | 3,51 |
| 2 | DBD | trauma capitis | 41 | 0 | 2,11 |
| 3 | DBD | trauma capitis | 18 | 2,85 | 0 |
| 4 | DBD | stroke | 44 | 0,94 | 0,46 |
| 5 | DCD | car accident, recuscitation | 21 | 0 | 3,9 |
| 6 | DCD | subarachnoid hemorrhage | 48 | 0,81 | 1,21 |
| 7 | DBD | subarachnoid hemorrhage | 51 | 0 | 1,85 |
| 8 | DCD | cardiac arrest | 66 | 0,27 | 0 |
| 9 | DCD | subarachnoid hemorrhage | 54 | 0 | 0 |
| 10 | DBD | cerebellar stroke | 74 | 1,02 | 2,13 |
| 11 | DCD | pulmonary embolisms, recuscitation | 30 | 0,18 | 0 |
| 12 | DCD | stroke | 62 | 0 | 1,81 |
| 13 | DCD | stroke | 58 | 0 | 1,29 |
| 14 | DCD | trauma capitis | 58 | 0 | 0,38 |
| 15 | DBD | carbonmonoxide intoxication, resusc. | 63 | 0 | - |
| 16 | DCD | trauma | 31 | 0,43 | 1,05 |
| 17 | DCD | stroke | 59 | 0 | 0,44 |
| 18 | DCD | stroke | 59 | 0,3 | 0 |
| 19 | DBD | trauma capitis | 21 | 0 | 0 |
| 20 | DBD | hemorrhagic stroke | 49 | 0,77 | 0 |
| 21 | DCD | complicated treatment for lungfibrosis | 65 | 0,25 | 0,26 |
| 22 | DCD | hemorrhagic stroke | 65 | 1,2 | 0 |
| 23 | DBD | subarachnoid hemorrhage | 50 | 0,36 | 0 |
| 24 | DCD | ischemic stroke, resuscitation | 51 | 0,55 | 2,18 |
| 25 | DBD | stroke | 57 | 0,7 | 0,61 |
| 26 | DBD | hemorrhagic stroke | 45 | 0 | 0 |
| 27 | DBD | subarachnoid hemorrhage | 67 | 0 | 0,38 |
| 28 | DBD | postanoxic encephalopathy after cardiac arrest | 49 | 0 | 0,8 |
